# FACTFINDERS for PATIENT SAFETY: Minimizing complications in radiofrequency neurotomy: Part I—Implantable devices; Part II—Preprocedural BVNRFN imaging

**DOI:** 10.1016/j.inpm.2025.100650

**Published:** 2025-11-22

**Authors:** David Hao, Gerald Yeung, Haewon Lee, Zheyan Chen, Ben Laplante, Paul Kitei, Reza Ehsanian, David Levi, International Pain and Spine Intervention Society's Patient Safety Committee, David Hao, Haewon Lee, Gerald Yeung, Zheyan Chen, David Levi, Gerald Yeung, Ben Laplante, David Hao, Paul Kitei, Reza Ehsanian, David Levi

**Affiliations:** a Massachusetts General Hospital, Boston, MA, USA; b Stanford University Medical Center, Stanford, CA, USA; c Jefferson Moss-Magee Rehab, Philadelphia, PA, USA; d Oregon Health and Science University, Portland, OR, USA; e University of Rochester Medical Center, Rochester, NY, USA; f Rothman Orthopaedic Institute, Philadelphia, PA, USA; g Sidney Kimmel Medical College, Thomas Jefferson University, Philadelphia, PA, USA; h University of New Mexico, Albuquerque, NM, USA; i Jordan-Young Institute, Virginia Beach, VA, USA

## Abstract

This series of FactFinders presents a brief summary of the evidence and outlines recommendations regarding minimizing risks with radiofrequency neurotomy procedures.

The evidence in support of the following facts is presented: (1) While data on the safety of radiofrequency neurotomy in patients with implanted devices are limited, the procedure can likely be performed safely with careful adherence to best practices and manufacturer recommendations. (2) Basivertebral nerve radiofrequency neurotomy can be performed safely and effectively at the L3 through S1 vertebral levels. Scrutiny of preprocedural imaging may minimize risk. Although the transpedicular approach is preferred, patient anatomy may dictate a non-transpedicular trajectory.

## Funding statement

No funding was utilized in the preparation of this manuscript.

HaoDavidLeeHaewonYeungGeraldChenZheyanLeviDavidon behalf of theon behalf of the International Pain and Spine Intervention Society's Patient Safety Committee

**FACTFINDERS FOR PATIENT SAFETY: Safety of Radiofrequency Neurotomy in Patients with Implanted Devices**

**Myth: Radiofrequency neurotomy is absolutely contraindicated in patients with implanted devices, such as those used for deep brain or spinal cord stimulation.**

**Fact: While data on the safety of radiofrequency neurotomy in patients with implanted devices are limited, the procedure can likely be performed safely with careful adherence to best practices and manufacturer recommendations.**

Radiofrequency neurotomy (RFN) is a minimally invasive intervention used in pain medicine for painful conditions, including pain arising from the facet joints [1]. RFN converts radiofrequency waves into thermal energy via an electrode, which heats surrounding tissue and coagulates the adjacent nerves. Thermal coagulation aims to disrupt nociceptive signals arising from targeted nerves supplying the facet joints or other structures.

Previous FactFinders have reviewed the safety of medial branch RFN in patients with a permanent pacemaker (PPM) or an implantable cardioverter defibrillator (ICD) and posterior spinal hardware [2,3]. Concern has also been raised about the safety of RFN in patients with implanted electrical devices such as those used for deep brain stimulation (DBS), spinal cord stimulation (SCS), or bladder, peripheral nerve, and vagus nerve stimulation.

1 **Deep brain stimulation**

DBS is FDA-approved for treating essential tremors, dystonia, Parkinson's disease, and treatment-resistant obsessive-compulsive disorder (OCD). It is also being explored as a treatment for other conditions, including depression and Alzheimer's disease. DBS operates on the principle of using a small electrode to deliver electrical impulses to specific brain regions. The standard electrode configuration is quadripolar, featuring four stimulating electrode contacts at the probe's tip. The implanted pulse generator (“battery”) is typically placed under the skin of the upper chest.

Theoretical risks associated with RFN include iatrogenic heating or stimulation of the device's battery or electrodes, which could occur during sensory and motor stimulation, as well as during the lesioning phase of the neurotomy protocol. Exposure to radiofrequency energy may cause unintended adverse effects on the implanted device or its leads by converted electrical or thermal energy [4]. Radiofrequency energy may also create magnetic fields, which could cause a stimulator device's spontaneous and unintended reactivation [5].

The primary literature on the safety of RFN in patients treated with DBS is limited to case reports and expert opinions. These sources generally integrate manufacturer recommendations and best practices for managing RFN in patients with pacemakers and ICDs [[5], [6], [7], [8]].

A 2009 case report by Osborne et al. described a patient with advanced Parkinson's disease requiring two implanted DBS devices who underwent lumbar medial branch RFN without complications [5]. The authors offered several recommendations, including setting the DBS stimulator voltage to zero and turning it off during the procedure, using bipolar RF to minimize the size of the induced electromagnetic field, gradually increasing voltage during sensory and motor stimulation, maintaining constant communication with the patient during the procedure, and comparing the post-procedure neurological exam with baseline measurements after the DBS stimulator is restored to its pre-procedural settings.

A second case report from 2020 involved a patient with Parkinson's disease who had a left subthalamic nucleus DBS device with the generator implanted in her right chest and underwent treatment for chronic low back pain [6]. The device was set to surgery mode, which deactivates stimulation, and a bipolar technique was employed during the ablation of the bilateral L3 and L4 medial branches and L5 dorsal rami. The patient tolerated the procedure well, and her post-procedural neurological exam remained unchanged. The authors noted adherence to recommendations from the Osborne case and highlighted that, unlike other manufacturers, this patient's device was an Abbott model, which required the use of surgery mode [5]. According to the authors' review of available online information and device manuals, Medtronic and Boston Scientific DBS devices currently do not offer special modes [6].

A third case report from 2023 involved a 78-year-old male with a DBS implanted for essential tremors [7]. The device manufacturer (Medtronic) stated that patients with implanted DBS were not recommended to undergo RFN due to the risk of device malfunction. However, the authors noted that there were previous cases of successful lumbar RFN in patients with DBS [5,6,9] and proceeded with safety precautions, including ensuring that the patient could tolerate symptoms of essential tremors in a trial of turning the DBS off for an hour-long period, stimulating the amount of time that the DBS would be turned off during an RFN; consulting the device representative, neurosurgeon, and neurologist regarding the risks and benefits of proceeding with an RFN; placing the ground pad contralateral to the DBS implantable pulse generator (IPG) and within 6 cm of the RFN cannula insertion sites to reduce current dispersion. The patient successfully tolerated a bilateral L3, L4 medial branch and L5 dorsal ramus unipolar RFN. The authors strongly cautioned against placing RFN cannulas less than 6 inches away from the DBS IPG when performing spine RFN to avoid electromagnetic interference on the DBS system, which can occur at close distances, even with proper placement of a dispersive electrode.

2 **Spinal cord stimulation**

SCS is a therapeutic modality used primarily to manage chronic pain conditions, including failed back surgery syndrome, complex regional pain syndrome, and refractory neuropathic pain. The SCS system consists of a pulse generator, typically implanted subcutaneously, and one or more electrode leads positioned in the epidural space.

The available literature on the safety of RFN in patients with SCS is also limited, primarily consisting of case reports and expert opinions.

A case report from 2010 details an incident involving a 49-year-old male patient who experienced spontaneous activation of a cervical spinal cord stimulator during RFN [10]. The patient previously underwent SCS placement, with one lead electrode positioned at the C4 midline and the other at C3 on the right. One year later, left TON and C3 RFN was attempted. Although the SCS was turned off, RF lesioning of the left third occipital nerve caused the patient to experience severe pain and paresthesia in both hands attributed to activation of the SCS system. While the RF probe's temperature was increasing to the targeted temperature for RFN, there was an unexpected and sudden increase from 40 °C to 60 °C within 10 s, necessitating the procedure's immediate cessation. The authors suggested that placing the RF cannula too close to the device could generate sufficient electromagnetic energy to interfere with the neurostimulator's function. Despite the incident, no issues with SCS functioning were found with the SCS thereafter, and the patient reported slight pain relief at a one-month follow-up. This case highlights the potential for RFN to interfere with SCS devices, even when deactivated. The authors suggest that the RF electromagnetic field may have induced unintended activation of the SCS. To mitigate this risk, it was recommended to maintain a safe distance between the RF cannula and SCS components, though no specific distance was specified.

A 2020 report highlights two cases of motor activation during lumbar RFN in both patients who had previously undergone bilateral S1 dorsal root ganglion (DRG) stimulation implantation [11]. In both cases, during the initiation of RFN, patients experienced leg discomfort and motor activation, prompting the procedure to be halted. It was later discovered that the stimulators had inadvertently been left in the "on" mode during the RFN procedure. In the first case, the dispersive pad was positioned on the left side of the patient's lower back during the right-sided RFN, 13.5 cm from the closest RF cannula. The IPG, in this case, was located 12.2 cm from the lower L5 RF cannula in the right buttock. In the second case, the grounding pad was placed contralaterally to the site of the RFN, but the distances between the grounding pad, IPG, and RF cannulae were not measured.

In both instances, after turning off the DRG-S systems, the RFN was successfully completed without complications, and both patients remained neurologically intact. At 5-month follow-up, both patients reported continued effective pain relief from their DRG-S systems, with no post-procedure issues.

The report suggested that the unintended S1 motor stimulation was likely due to the effect of RF energy on the DRG-S leads. Several factors contributed to this phenomenon, including the proximity of the DRG-S leads to the RF cannulae and the use of monopolar RF, which allows for a broader energy path. To mitigate these risks, the authors recommended using bipolar RF techniques, which confine the flow of RF energy between the active tips of the cannulae, thereby reducing the risk of interference. Additionally, activating the "surgery mode" on the SCS device, if available, can help prevent device malfunction by releasing a small amount of current to block the lead from becoming a conductor for the RF energy. Ensuring that the distance from the RF cannulae to the dispersive pad is shorter than the distance to the SCS pulse generator or leads was also advised to minimize the risk of interference.

A retrospective cohort analysis published in 2014 evaluated the safety of RFN in patients with implanted medical devices, including SCS, pacemakers, and ICDs [12]. Over five years, 30 patients underwent 68 RFN procedures targeting lumbar facet joints or sacroiliac joints. The study included a thorough pre-procedural evaluation of each implanted device, focusing on parameters like stimulus frequency, pulse duration, and electrode impedance for SCS. RFN was conducted under local anesthesia, allowing patients to report any issues immediately. The procedures were safely performed in all patients with no reported incidents of electrical interference or device malfunction. Post-procedure evaluations confirmed that RF treatments did not adversely affect any devices.

3 **Bladder stimulators, peripheral nerve stimulator, and vagus nerve stimulators**

The authors are unaware of any literature related to RFN in the presence of bladder stimulators, peripheral nerve stimulators, or vagus nerve stimulators.

4 **Conclusions/recommendations**

● Before RFN in patients with implanted devices, discuss potential risks with the patient, including the possibility of device interference or malfunction.
● Ensure careful monitoring for interference or malfunction, and maintain continuous communication with the patient to promptly address any abnormal sensations or issues.
● If utilized, minimal conscious sedation is advised.
● To mitigate the risk of interference caused by the radiofrequency energy, consider using bipolar RF techniques (with proper training), positioning the dispersive pad closer to the RF target than to the implanted devices or its leads, and activating “surgery mode” if available.
● Immediately after the procedure, reactivate the implanted device or turn off “surgery mode” and confirm the device's functionality to ensure continued therapeutic efficacy and patient safety.
● There is limited literature on RFN in patients with bladder or peripheral nerve stimulators. We recommend consulting the manufacturer's guidelines; if these are unavailable or insufficient, contacting the implanting provider of the pre-existing device can help inform the shared decision-making process and the discussion of risks and benefits with the patient.

References1DreyfussP.HalbrookB.PauzaK.JoshiA.McLartyJ.BogdukN.Efficacy and validity of radiofrequency neurotomy for chronic lumbar zygapophysial joint painSpine (Phila Pa 1976)251020001270127710.1097/00007632-200005150-00012108065052SmithC.DeFranceschF.PatelJ.Spine Intervention Society’s Patient Safety CommitteeRadiofrequency neurotomy for facet joint pain in patients with permanent pacemakers and defibrillatorsPain Med202201941141210.1093/pm/pny213303588653SaffarianM.ChristoliasG.BabariaV.FactFinders for patient safety: motor stimulation testing in lumbar radiofrequency neurotomy and radiofrequency neurotomy in patients with posterior hardwareInterv Pain Med21202310017010.1016/j.inpm.2022.100170Published 2023 Mar 28PMC11372923392396094RahimpourS.KiyaniM.HodgesS.E.TurnerD.A.Deep brain stimulation and electromagnetic interferenceClin Neurol Neurosurg203202110657710.1016/j.clineuro.2021.106577PMC8081063336627435OsborneM.D.Radiofrequency neurotomy for a patient with deep brain stimulators: proposed safety guidelinesPain Med1062009 Sep1046104910.1111/j.1526-4637.2009.00686.x197442166AngS.P.MaddaloS.DoanL.V.Lumbar medial branch radiofrequency neurotomy in a patient with deep brain stimulatorPain Med Case Rep4520201751787BanikR.K.PengS.KohanL.RanaP.DarrowD.P.HagedornJ.M.Radiofrequency ablation of lumbar medial branch nerves in a patient with a deep brain stimulator: our experience and literature reviewPain Med246202373473710.1093/pm/pnac179363777778SowderT.SayedD.ConcannonT.The American society of pain and neuroscience (ASPN) guidelines for radiofrequency ablative procedures in patients with implanted devicesJ Pain Res1620233693370610.2147/JPR.S419594Published 2023 Nov 337942223PMC106295079DoanL.V.Case report: lumbar medial branch radiofrequency neurotomy in a patient with deep brain stimulatorPain Manag Case Rep45202017517810JeonH.Y.ShinJ.W.KimD.H.SuhJ.H.LeemJ.G.Spinal cord stimulator malfunction caused by radiofrequency neuroablation -A case report-Korean J Anesthesiol59Suppl2010S226S22810.4097/kjae.2010.59.S.S226Suppl21286447PMC303004311ChapmanK.B.SchirripaF.YousefT.DeygooJ.van HelmondN.Lumbar radiofrequency ablation interfering with S1 dorsal root ganglion stimulation systems: experience from two casesPain Pract207202078078610.1111/papr.129013230647312BarbieriM.BelliniM.Radiofrequency neurotomy for the treatment of chronic pain: interference with implantable medical devicesAnaesthesiol Intensive Ther463201416216510.5603/AIT.2014.002925078768

YeungGeraldLaplanteBenHaoDavidKiteiPaulEhsanianRezaLeviDavidon behalf of theon behalf of the International Pain and Spine Intervention Society's Patient Safety Committee

**FACTFINDERS FOR PATIENT SAFETY: Minimizing Risk with Basivertebral Nerve Radiofrequency Neurotomy: Preprocedural Imaging Recommendations**

**Myth: Basivertebral nerve radiofrequency neurotomy (BVNRFN) can be performed safely and effectively at all levels in a standardized fashion using only a transpedicular approach.**

**Fact: BVNRFN can be performed safely and effectively at the L3 through S1 vertebral levels. Scrutiny of preprocedural imaging may minimize risk. Although the transpedicular approach is preferred, patient anatomy may dictate a non-transpedicular trajectory.**

BVNRFN selectively disrupts pain signaling from vertebral endplates to treat vertebrogenic chronic axial back pain with concurrent Modic type 1 or type 2 changes on MRI. BVNRFN involves accessing the pedicle, introducing a curved cannula to create a channel, inserting a radiofrequency probe, and ablating the basivertebral nerve [1]. An extrapedicular/parapedicular trajectory for vertebral body access has been described and may be considered depending on pedicular width or presence of pedicle screws [2]. While the reported rate of complications is low with both transpedicular and extrapedicular approaches, those described to date include nerve root injury and radicular pain and/or neurological deficit, incisional pain, urinary retention, lumbar and sacral fractures, retroperitoneal hemorrhage, hemorrhage posterior to the vertebral body, new-onset back pain in a different location, and lateral femoral cutaneous neuropraxia [1,[3], [4], [5], [6], [7]].

Minimizing the risk of lumbar artery, segmental artery, and nerve root injury involves avoiding direct vascular or neural contact, minimizing thermal injury to nerve root with final probe placement, preventing pedicle breach or fracture, and reducing the likelihood of vertebral body compromise. Scrutiny of available MRI and CT imaging can aid in preprocedural planning to minimize neurovascular injury.

1 **Vascular**

Amongst the most serious complications associated with BVNRFN is vascular injury, which carries the potential for subsequent hemorrhage and shock. In the literature, this has been documented once due to misdirected pedicle access [1,[3], [4], [5], [6]]. An excessively lateral position resulted in a psoas hematoma, and the resulting compressive lesion caused transient neuropraxia on the femoral nerve [3]. An additional case of a hematoma was reported posterior to the vertebral body with tenting of the posterior longitudinal ligament [7]. However, lumbar artery iatrogenic injury has been described in the literature with other percutaneous vertebral access procedures (vertebroplasty/kyphoplasty) with both transpedicular and extrapedicular puncture [8]. A thorough review of available imaging can focus on visualizing the pertinent vascular structures to avoid when planning the trajectory of the introducer trochar. The lumbar arteries originate from the abdominal aorta, typically distributing in pairs from the L1 to L4 levels, and rarely from the L5 level. They then traverse to the posterolateral side of the vertebral body, dividing into three branches (anterior, posterior, and middle branches) anterior to the neuroforamen. However, significant anatomic variation may occur, including differences in size and width, the number of arteries present, whether a common trunk is present, variations in branching patterns, and differences in their anatomical course [[8], [9], [10], [11]].

2 **Neural**

Spinal cord injury would potentially also be amongst the most serious complications that may be associated with BVNRFN. Given the termination of the spinal cord above levels usually targeted for BVNRFN with on-label use (L3-S1), direct spinal cord trauma has not been reported. However, spinal cord injury has been associated with other vertebral body access procedures and may be secondary to epidural hematoma [12].

The most frequently reported adverse events include transient motor/sensory disturbances and/or radiculopathy [1,[3], [4], [5], [6]]. As mentioned above, it is important to review and consider the pertinent neural anatomy to minimize direct contact with pedicular breach and/or thermal injury during final probe placement when performing the ablation portion of the procedure. The lumbar nerve roots run near the pedicles and vertebral bodies, with the pedicles forming the roof and floor of the neuroforamen, and the dorsal root ganglion often lying directly beneath the pedicles [13,14]. More specifically, the traversing nerve root lies in the lateral recess just medial to the pedicle, and the exiting nerve root runs just inferior to the mid-portion of the pedicle within the superior aspect of the foramen. Given the anatomical structures abutting the pedicles, careful evaluation of pedicular morphology is essential to minimize the risk of pedicular breach during the transpedicular approach.

3 **Pedicular breach**

To minimize pedicular breach, evaluate the pedicle diameters and access the larger of the two pedicles available at any given vertebral body level if one is too small to access with the available instrument. In the spine surgery literature, an 80 % pedicle screw diameter-to-pedicle width ratio is generally considered a conservative threshold to reduce the risk of pedicle breach, so consider the size of the pedicle to the pedicular access instrument used in basivertebral nerve ablation. It is additionally recommended to maintain a minimum cortical margin of 0.5 mm on both the medial and lateral aspects of the pedicle to minimize the risk of pedicle fracture [15]. However, in the spine surgical literature, it is known that transpedicular intervention can reduce the axial resistance force of the pedicle, which may lead to pedicle fracture [16]. While the risk of fracture may not be the same for proceduralists with a single trochar compared to maintaining margin for placing pedicle screws, when considering pedicular access versus extrapedicular vertebral body access, proceduralists should carefully determine which side of the pedicle offers the greatest safety margin for trochar insertion based on width and cortical margin. One may consider extrapedicular access when pedicle width is inadequate for the trochar on either side, when there are preexisting pedicle screws, or when there is presence of vasculature in the trajectory with pedicular access [2]. Thus, it is important to scrutinize MRI for pedicular width, prior instrumentation, and neurovasculature. However, it is important to consider the limitations of MRI. A comparative observational study comparing MRI and CT in the lumbar spine found that pedicle diameters on CT were 0.4–0.5 mm wider than on MRI [17]. Thus, if MRI shows adequate pedicle diameter, further imaging is not required. However, if there is any doubt about whether the pedicle can accommodate the trochar, obtaining a CT scan could be considered to evaluate bony anatomy best when planning for pedicle access [18]. Otherwise, this may be a time to defer to an extrapedicular approach to avoid the possibility of accessing a pedicle without adequate width.

Pedicle transverse angulation and width typically increase from L1 to L5 [19]. Ideally, the C-arm should be obliqued to at least 30° to allow the trochar to enter the pedicle and vertebral body at an angle that facilitates the accurate placement of the radiofrequency probe. However, the precise C-arm angle required varies depending on individual anatomical variations. Some patients may have pedicles oriented in the sagittal plane and/or exhibit extremely narrow pedicles at mid-to-upper lumbar levels. In patients with markedly sagittal pedicle orientation, accurate probe placement can be challenging, increasing the risk of procedural failure due to anterior radiofrequency probe placement. Conversely, in patients with very narrow pedicles, there is an elevated risk of pedicular breach or fracture. When pre-procedural imaging reveals a pedicle angulation significantly less than 30°, an off-label parapedicular or extra-pedicular approach may be considered.

Access to the S1 segment is typically achieved using a Ferguson view (with the S1 superior endplate squared off). Iliac crest anatomy can complicate S1 pedicular access, particularly in male patients with high and narrow iliac crests. Pre-procedural X-ray imaging can assist the interventionalist in selecting the pedicle that allows for a more favorable oblique trajectory. In patients whose anatomy would otherwise result in excessively ventral probe placement, increasing the rostral tilt beyond the Ferguson view may permit greater obliquity. However, care must be taken to avoid excessively caudal positioning of the instrument within the vertebral body in such scenarios.

The lumbar basivertebral nerve is generally located at the midpoint of the vertebral body, between the superior and inferior endplates [20], whereas the S1 basivertebral nerve is often situated slightly closer to the superior endplate [21]. Nonetheless, significant anatomical variability exists at S1, particularly in patients with transitional anatomy. Although the basivertebral nerve itself cannot be directly visualized, it travels with the basivertebral vessels [20], which are often visible on sagittal MRI. A thorough review of imaging is essential to account for individual anatomical differences and to try to ensure accurate lesioning of the basivertebral nerve.

4 **Fracture**

BVNRFN has not been strongly associated with long-term adverse effects on the vertebral body in the initial trials with normal bone density study patients [22]. However, BVNRFN may transiently weaken the bone in patients with existing osteoporosis, with vertebral compression fractures (VCFs) presenting on average two months after the procedure. VCFs in the SMART Trial (n = 225) were found to have an exceedingly low prevalence of 0.4 %; however, the study excluded patients with scoliosis, spondylolisthesis, and osteoporosis [3]. Notably, the lone fracture was in a sham patient who crossed over to active treatment at one year and was only found to have osteopenia on subsequent evaluation. A subsequent observational study (n = 74) without the previously noted exclusions found a higher incidence of VCFs at 12 %, at an average interval of 69 days following BVNRFN [23]. Of note, the mean age of these patients with VCFs was 78 years, and all of the patients with VCFs had osteoporosis with a mean T-score of −3.0. Thus, interventionalists should consider reviewing T-scores in patients with advanced age. For those with significant osteoporosis, it is important to have a risk-benefit discussion regarding BVNRFN and VCF risk.

5 **Summary**

● A review of MRI for pedicle width, pedicle orientation, and basivertebral nerve location is recommended.
● MRI may underestimate pedicle width; therefore, if there is any doubt about whether the pedicle width can accommodate the trochar, consider CT imaging.
● While off-label with Intracept, a parapedicular or extrapedicular approach with BVNRFN could be considered when the pedicle is too narrow to minimize the risk of pedicle breach or if the pedicles are too sagittally oriented to avoid a final probe position that is too ventral.
● When pursuing a parapedicular approach, it is crucial to review the locations of the lumbar artery and segmental arteries to minimize the risk of vascular injury.
● Reviewing T-score in patients with advanced age and having a risk-benefit discussion regarding BVNRFN and VCF risk for patients with significant osteoporosis are recommended.

References1CongerA.SchusterN.M.ChengD.S.SperryB.P.JoshiA.B.HaringR.S.The effectiveness of intraosseous basivertebral nerve radiofrequency neurotomy for the treatment of chronic low back pain in patients with modic changes: a systematic reviewPain Med2252021 May 211039105410.1093/pm/pnab040PMID: 33544851335448512StolzenbergD.KiteiP.M.TranD.PfeiferR.Intracept technique at adjacent levels to fusions with pedicle screwsInterv Pain Med222023 Jun 1410026110.1016/j.inpm.2023.100261PMID: 39238666; PMCID: PMC11372909PMC11372909392386663FischgrundJ.S.RhyneA.L.FrankeJ.SassoR.C.KitchelS.H.BaeH.W.Intraosseous basivertebral nerve ablation for the treatment of chronic low back pain: 2-Year results from a prospective randomized double-blind sham-controlled multicenter studyInt J Spine Surg1322019 Apr 3011011910.14444/6015PMID: 31131209; PMCID: PMC651018031131209PMC65101804KhalilJ.G.SmuckM.KoreckijT.KeelJ.BeallD.GoodmanB.A prospective, randomized, multicenter study of intraosseous basivertebral nerve ablation for the treatment of chronic low back painSpine J19102019 Oct1620163210.1016/j.spinee.2019.05.598Epub 2019 Jun 20. PMID: 31229663312296635BeckerS.HadjipavlouA.HeggenessM.H.Ablation of the basivertebral nerve for treatment of back pain: a clinical studySpine J1722017 Feb21822310.1016/j.spinee.2016.08.032Epub 2016 Sep 1. PMID: 27592808275928086TruumeesE.MacadaegK.PenaE.ArbuckleJ.GentileJ.FunkR.A prospective, open-label, single-arm, multi-center study of intraosseous basivertebral nerve ablation for the treatment of chronic low back painEur Spine J2872019 Jul1594160210.1007/s00586-019-05995-2Epub 2019 May 21. PMID: 31115683311156837FoxS.LevinJ.Hematoma formation after basivertebral nerve ablationInterv Pain Med432025 Jul 3010061710.1016/j.inpm.2025.100617PMID: 40786847; PMCID: PMC12332941PMC12332941407868478LiuL.LiN.WangQ.WangH.WuY.JinW.Iatrogenic lumbar artery injury in spine surgery: a literature reviewWorld Neurosurg1222019 Feb26627110.1016/j.wneu.2018.10.219Epub 2018 Nov 9. PMID: 30419401304194019LiuL.ChengS.WangQ.LiangQ.LiangY.JinW.An anatomical study on lumbar arteries related to the extrapedicular approach applied during lumbar PVP (PKP)PLoS One1432019 Mar 5e021316410.1371/journal.pone.0213164PMID: 30835754; PMCID: PMC6400376PMC64003763083575410IwamotoS.TakaoS.HaradaM.Visualization of lumbar artery variations by contrast-enhanced multi-detector row computed tomographyJ Med Investig631–22016454810.2152/jmi.63.45PMID: 270400512704005111ChoS.M.NamY.S.ChoB.M.LeeS.Y.OhS.M.KimM.K.Unilateral extrapedicular vertebroplasty and kyphoplasty in lumbar compression fractures: technique, anatomy and preliminary resultsJ Korean Neurosurg Soc4952011 May27327710.3340/jkns.2011.49.5.273Epub 2011 May 31. PMID: 21716899; PMCID: PMC311514721716899PMC311514712FangM.ZhouJ.YangD.HeY.XuY.LiuX.Management and outcomes of spinal epidural hematoma during vertebroplastyMedicine (Baltim)97212018 Maye1073210.1097/MD.0000000000010732PMID: 29794750; PMCID: PMC6393141PMC63931412979475013EbraheimN.A.XuR.DarwichM.YeastingR.A.Anatomic relations between the lumbar pedicle and the adjacent neural structuresSpine (Phila Pa 1976)22201997 Oct 152338234110.1097/00007632-199710150-00003PMID: 9355213935521314CohenM.S.WallE.J.BrownR.A.RydevikB.GarfinS.R.1990 AcroMed award in basic science. Cauda equina anatomy. II: extrathecal nerve roots and dorsal root gangliaSpine (Phila Pa 1976)15121990 Dec12481251PMID: 2281367228136715SolitroG.F.WhitlockK.AmiroucheF.MehtaA.I.McDonnellA.Currently adopted criteria for pedicle screw diameter selectionInt J Spine Surg1322019 Apr 3013214510.14444/6018PMID: 31131212; PMCID: PMC651017831131212PMC651017816MeisterhansM.HagelV.SpirigJ.M.FasserM.R.FarshadM.WidmerJ.The biomechanics of the transpedicular endoscopic approachSpine (Phila Pa 1976)49152024 Aug 11052105810.1097/BRS.0000000000004871Epub 2023 Nov 7. PMID: 37942817; PMCID: PMC1123294037942817PMC1123294017PachaT.O.OmarM.GraulichT.SueroE.Mathis SchrÖderB.KrettekC.StubigT.Comparison of preoperative pedicle screw measurement between computed tomography and magnet resonance imagingInt J Spine Surg1452020 Oct67168010.14444/7098Epub 2020 Oct 23. PMID: 33097582; PMCID: PMC767144233097582PMC767144218SarwahiV.AmaralT.WendolowskiS.GecelterR.SugarmanE.LoY.MRIs are less accurate tools for the Most critically worrisome pedicles compared to CT scansSpine Deform462016 Nov40040610.1016/j.jspd.2016.08.002Epub 2016 Oct 26. PMID: 279275682792756819GoyalD.K.C.TarazonaD.A.SegarA.SuttonR.MottoM.A.DiviS.N.Lumbar pedicle morphology and vertebral dimensions in isthmic and degenerative Spondylolisthesis—A comparative studyInt J Spine Surg1522021 Apr24325010.14444/8009Epub 2021 Feb 1833900981PMC805938020BaileyJ.F.LiebenbergE.DegmetichS.LotzJ.C.Innervation patterns of PGP 9.5-positive nerve fibers within the human lumbar vertebraJ Anat21832011 Mar26327010.1111/j.1469-7580.2010.01332.xEpub 2011 Jan 12. PMID: 21223256; PMCID: PMC305821221223256PMC305821221DegmetichS.BaileyJ.F.LiebenbergE.LotzJ.C.Neural innervation patterns in the sacral vertebral bodyEur Spine J2562016 Jun1932193810.1007/s00586-015-4037-4Epub 2015 Jun 16. PMID: 26077098; PMCID: PMC468169826077098PMC468169822LorioM.Clerk-LamaliceOlivierBeallD.P.JulienT.International society for the advancement of spine surgery Guideline—Intraosseous ablation of the basivertebral nerve for the relief of chronic low back painInt J Spine Surg1412020 Feb 29182510.14444/7002PMID: 32128298; PMCID: PMC704383532128298PMC704383523FogelG.MusieJ.PhillipsT.R.ShonnardM.YoussefS.HirschJ.A.Assessment and management of patients developing low energy vertebral compression fractures following basivertebral nerve ablationPain Med2532024 Mar 124925110.1093/pm/pnad132PMID: 3775670137756701

## Declaration of competing interest

None.
